# Effects of conditioning, source, and rest on indicators of stress in beef cattle transported by road

**DOI:** 10.1371/journal.pone.0244854

**Published:** 2021-01-12

**Authors:** Daniela M. Meléndez, Sonia Marti, Derek B. Haley, Timothy D. Schwinghamer, Karen S. Schwartzkopf-Genswein

**Affiliations:** 1 Lethbridge Research and Development Centre, Agriculture and Agri-Food Canada, Lethbridge, Alberta, Canada; 2 Department of Ruminant Production, IRTA, Caldes de Montbui, Barcelona, Spain; 3 Department of Population Medicine, University of Guelph, Guelph, Ontario, Canada; Michigan State University, UNITED STATES

## Abstract

Consumers are increasingly concerned about the welfare of farm animals, especially during transportation. The Canadian transport regulations state that weaned cattle require an 8 h rest after 36 h of transport. There are, however, only a few studies that assess the effect of rest on the welfare of beef cattle. The aim of this study was to assess the effect of conditioning, source and rest on indicators of welfare in 7-8-month-old beef calves during long-distance transport. Treatments consisted of a 2 × 2 × 2 factorial design where the main factors were conditioning: conditioned (**C**; *n* = 160) or non-conditioned (**N**; *n* = 160); source: auction market (**A**; *n* = 160) or ranch direct (**R**; *n* = 160); and rest: 0 h (**0 h**; *n* = 160) or 8 h (**8 h**; *n* = 160). Means of non-esterified fatty acids (NEFA), serum amyloid-A, haptoglobin, creatine kinase (CK), and percentage of time standing from N calves were greater than C calves (all *p* ≤ 0.05). Means of percentage of time standing and CK of R calves were greater than A calves (both *p* ≤ 0.05). The mean of NEFA of 0 h calves was greater than the 8 h calves (*p* < 0.01), while the percentage of time standing of 0 h calves was less than 8 h calves (*p* < 0.01). Statistically significant differences between means of NEFA and standing percentage, were observed between 0 h and 8h calves. Few and inconsistent indicators of reduced welfare were observed between auction market and ranch direct calves, while non-conditioning was associated with greater physiological and behavioural indicators of reduced welfare. Based on these results, conditioning should be implemented as a management practice to improve the welfare of transported calves.

## Introduction

Consumers are increasingly concerned about the welfare of farm animals being raised for food consumption [1]. A study assessing the perception of consumers through focus groups and interviews reported that transport is of particular concern to consumers, even more than painful husbandry procedures, due to the high visibility of livestock transportation in urban areas [2]. Livestock trailers in North America are generally not equipped with feeders or drinkers, and cattle are unable to lie down during transportation. Providing a rest stop could improve animal welfare, as it provides an opportunity for animals to eat, drink and lie down, however, it could also increase the risk of disease due to exposure to pathogens and increased stress associated with mixing with other cattle and additional animal handling [3]. Amendments to the Canadian humane transport regulations came into effect in February of 2020, reducing the maximum time in transit for weaned cattle from 48 to 36 h before a mandatory rest stop, which was extended from 5 to 8 h [4]. There are, however, few and contradictory studies that assess the effect of rest stops on beef cattle welfare. For example, improved welfare was reported in preconditioned calves that received a 2 h rest [5], while no rest or 5 h of rest was more beneficial than providing 10 or 15 h of rest in newly weaned calves [6], and no differences were observed between conditioned calves that received 0, 4, 8 or 12 h of rest [7].

Transport and weaning are stressful management procedures, which have been found to affect the immune response [8]. Preconditioning is a management practice that reduces stress by performing husbandry procedures (weaning, dehorning, castration, branding, ear tagging, vaccination, and adapting calves to a grain feed diet, the feed bunk and water trough) 30 to 45 d prior to transport [9, 10]. Preconditioned calves are characterized by reduced bovine respiratory disease related morbidity at the feedlot compared to non-preconditioned calves [11–13]. In a previous study assessing the effect of rest duration, no differences were observed in morbidity, mortality and certain welfare indicators of conditioned calves rested for 0, 4, 8 or 12 h [7]. Preconditioned calves may be more resilient to transportation related stress, and therefore may not benefit from a rest. Conversely, calves that are exposed to multiple stressors prior to transport, such as newly weaned calves, may benefit from a rest stop.

Reducing stressors associated with the marketing of feeder calves is known to reduce the risk of disease in beef cattle [14, 15]. Calves are sold most commonly to backgrounding or feedlot operations through auction markets, however the use of video or direct sales is increasing gradually [16]. Auction markets offer the opportunity to sell small lots of cattle, but have the disadvantage of contributing to calf weight loss (shrink), likely due to transport and limited or no access to feed, and a higher risk of disease due to comingling with other calves and limited biosecurity [17]. Video and direct sales are a convenient option for buyers and sellers, as it minimises shrink and the exposure to pathogens by shipping calves from the ranch of origin directly to the feedlot operation. Lower risk of developing bacterial bronchopneumonia has been reported in calves purchased directly from the ranch than calves purchased through the auction markets [14, 15]. Similar to newly-weaned calves, auction market calves may benefit from a rest stop because they are exposed to multiple stressors close to the time of transportation compared to calves sourced directly from a ranch.

Currently, there is a lack of literature on the effect of a rest stop on the welfare of beef cattle purchased from different sources and different management strategies. Therefore, the aim of this study was to assess the effect of conditioning, source, and rest, on welfare indicators in 7-8-month-old beef cattle transported by road.

## Materials and methods

This protocol was approved by the Animal Care Committee of Lethbridge Research and Development Centre (LeRDC) (ACC number 1918). Animals were cared for in accordance with the Canadian Council of Animal Care [18].

### Animal management and transport

Three hundred and twenty crossbred steer calves—Black or Red Angus × Hereford/Simmental and Black or Red Angus × Charolais, 245 ± 35.7 kg (mean ± SD) of body weight (BW)—were sourced from two different locations in southern Alberta, Canada. Treatments consisted of a 2 × 2 × 2 factorial design where the main factors included conditioning: conditioned (**C**; *n* = 160) or non-conditioned (**N**; *n* = 160); source: auction market (**A**; *n* = 160) or ranch direct (**R**; *n* = 160); and rest: 0 h (**0 h**; *n* = 160) or 8 h (**8 h**; *n* = 160). Calves were divided into two groups (group 1 and 2) and each group was transported by road for 36 h, rested, and transported for an additional 4 h, 8 d apart (Table 1). Samples were collected prior to loading (LO1) and after unloading (UN1) following the 36-h transport, as well as prior to loading (LO2) and after unloading (UN2) following the additional 4-h transport. In addition, calves were sampled on d 1, 2, 3, 5, 14 and 28 after UN2. Calves were randomly assigned to treatments (40 calves/treatment) and pens (10 calves/pen). To avoid variation in physiological parameters due to the circadian rhythm, calves were sampled 24 (1 d), 48 (2 d), and 72 (3 d) h after UN2.

**Table 1:** Chronology of sampling for group 1 and 2 of conditioned and non-conditioned, crossbred beef calves, sourced from an auction or ranch, transported for 36 h and rested for 0 or 8 h.

Samples	Group 1		Group 2	
	A	R	A	R
LO1	Sep 23^rd^ 1525–1735	Sep 24^th^ 1527–1726	Oct 1^st^ 1615–1753	Oct 2^nd^ 1612–1747
	**0 h**	**8 h**	**0 h**	**8 h**
UN1	Sep 26^th^ 0558–0848		Oct 4^th^ 0605–0901	
LO2	-	Sep 26^th^ 1702–1813	-	Oct 4^th^ 1700–18004
UN2	Sep 26^th^ 1131–1253	Sep 26^th^ 2227–0011	Oct 4^th^ 1154–1311	Oct 4^th^ 2217–2342
1 d	Sep 27^th^ 1129–1252	Sep 27^th^ 2224–2351	Oct 5^th^ 1136–1305	Oct 5^th^ 2214–2345
2 d	Sep 28^th^ 1130–1248	Sep 28^th^ 2225–2343	Oct 6^th^ 1140–1255	Oct 6^th^ 2210–2337
3 d	Sep 29^th^ 1127–1256	Sep 29^th^ *	Oct 7^th^ 1137–1300	Oct 7^th^ 2212–2330
5 d	Oct 1^st^ 0920–1150		Oct 9^th^ 0800–1049	
14 d	Oct 10^th^ 0802–1040		Oct 18^th^ 0855–1128	
28 d	Oct 24^th^ 0802–1038		Nov 1^st^ 0802–1046	

Values indicate the date and time (24 h clock) sampling took place.

Calves were sampled before (LO1) and after (UN1) the 36h transport, before (LO2) and after (LO2) the additional 4 h transport, as well as on d 1, 2, 3, 5, 14, and 28 after UN2. Calves were sourced from an auction market (A) or ranch (R) and were provided with either no rest (0 h) or 8 h (8 h) of rest.

*Calves rested for 8 h in group 1 were not sampled on Sep 29^th^ due to a severe snowstorm.

#### Conditioned calves

Twenty to twenty one d prior to LO1 (September 3^rd^ and 11^th^, 2019), two groups of eighty calves were weaned and transported for approximately 1 h from the ranch of origin to the LeRDC. Upon arrival calves were processed, which included receiving a 7-way bovine clostridial vaccine (Ultrabac/Somubac, Zoetis Canada Inc., Kirkland, QC, Canada); a 5-way bovine viral diarrhea, rhinotracheitis, parainfluenza and bovine respiratory syncytial virus vaccine (Pyramid FP 5 + Presponse SQ, Boehringer Ingelheim., Burlington, ON, Canada); an antibiotic (Draxxin, Zoetis Canada Inc., Kirkland, QC, Canada); an anti-parasitic agent (Ivomec Pour-on for Cattle, Boehringer Ingelheim, Burlington, ON, Canada); and an ear tag and a half duplex RFID tag. During the conditioning period (20 to 21 d) calves were housed in 4 pens (36.7 m × 22.2 m) with a central water trough, with 40 animals per pen. Calves received an *ad libitum* diet consisting of 35% corn silage, 20% alfalfa hay, 12% barley grain and 3% supplement with vitamins and minerals the first 3 d after arrival to the LeRDC feedlot and *ad libitum* feed consisting of 85% corn silage, 12% barley grain and 3% supplement with vitamins and minerals for the rest of the conditioning and experimental period.

In order for calves to be “preconditioned”, they must be weaned, castrated, dehorned, branded, vaccinated, ear tagged, and adapted to a grain diet, the feed bunk and/or the water trough for 30 to 45 d prior to transportation [9, 10]. In the present study, half of the calves were weaned, vaccinated, ear tagged, and adapted to the feed, the feed bunk, and the water trough for a period of 20 to 21 d prior to transport. Therefore, calves in this study will be referred to as ‘conditioned’ calves, because the suggested preconditioning time period prior to shipping was not met. A conditioning period of 20 to 21 days was selected to match the methods of a previous transport study that reported no differences in welfare indicators of rested and unrested calves [7].

#### Non-conditioned calves

Prior to LO1 (September 23^rd^ and 24^th^, and October 1^st^ and 2^nd^, 2019), four groups of forty calves were separated from their dams and put on a truck and transported for approximately 1 h from the ranch of origin to the LeRDC. Non-conditioned calves received an ear tag and an RFID tag during LO1 sampling for identification. Processing (vaccine, antibiotic, and anti-parasitic administration) was postponed until UN2 to simulate industry practices, where calves are processed once they arrived at the feedlot.

Upon arrival to the LeRDC, each group of 40 non-conditioned calves was mixed with 40 conditioned calves (housed at the LeRDC) prior to LO1 sampling. After LO1 and LO2 sampling, calves were sorted into 5 pens, in order for treatments to be equally distributed into one of the 5 compartments within the trailer.

#### Auction market calves

Conditioned (*n* = 80) and non-conditioned (*n* = 80) calves from the groups described above were sampled (LO1) and transported for approximately 20 min (September 23^rd^ and October 1^st^, 2019) to a local auction market 7.9 km from the LeRDC, off-loaded and sorted into pens with access to hay and water. Within the 24 h that calves spent at the auction market, they were moved through the sale ring to mimic auction market conditions. Although it is common for calves to be mixed at the auction market, calves in the present study were not comingled with calves from other farms due to time and labour limitations.

#### Ranch-direct calves

The remaining non-conditioned (*n* = 80) calves and conditioned (*n* = 80) calves were mixed and sampled at LO1 on (September 24^th^ and October 2^nd^, 2019) prior to the 36 h transport.

Auction market calves were transported to the auction market one day prior to the 36 h transport so that both auction market and ranch calves from each group were transported at the same time. Two separate trucks loaded auction market calves and ranch calves (LeRDC) at 1800 h. Calves did not have access to feed and water for 2.5 h prior to LO1, and for 1 h prior to LO2.

#### Housing and feeding

Calves were housed in 32 pens (21 × 27 m) with a fence line water through; 10 animals per pen. Calves received an *ad libitum* diet consisting of 35% corn silage, 20% alfalfa hay, 12% barley grain, and 3% supplement with vitamins and minerals for the first 3 d after arrival to the LeRDC feedlot. For the remainder of the conditioning and the experimental period, calves received *ad libitum* feed consisting of 85% corn silage, 12% barley grain and 3% supplement with vitamins and minerals to meet beef cattle nutrition requirements [19]. In addition, water was provided *ad libitum* through a central water trough in the conditioning pens, and through a fence line water trough in the experimental pens.

#### Transport

Two model 379 Peterbilt trucks and 2018 Merritt feeder cattle tri-axle trailers bedded with wood shavings were used to transport calves for 36 h and for an additional 4 h after rest. Calves transported for 36 h left the auction market and the LeRDC at 1800 h (September 24^th^ and October 2^nd^, 2019), and both trailers arrived to the LeRDC at 0600 h (September 26^th^ and October 4^th^, 2019). During both transports, calves were placed in the nose (*n* = 5), the deck (*n* = 26), the belly (*n* = 26), the back (*n* = 13), and the doghouse (*n* = 10). Loading densities (nose 1.63, deck 0.71, belly 0.71, back 0.91, and doghouse 1.18 m^2^/animal) differed between compartments in order to have an equal representation of treatments per compartment. The two trailers leaving the auction market and the LeRDC on the same day travelled together to ensure similar road and environmental conditions. Drivers monitored the calves when they stopped for rest and made sure that all calves were standing to avoid injuries. The same experienced drivers transported both group 1 and group 2 calves.

Trailer temperature and humidity were recorded using the DS1923 hygrochron temperature/humidity logger iButton (Maxim Integrated Products, Sunnyvale, CA, USA). Loggers were zip tied to the front of the ear tags of a subset of 128 calves, which were equally distributed by compartment. Relative humidity (RH, %) and temperature (T,°C) data were collected every 2 minutes during the 36 and 4 h transport and were used to calculate the temperature humidity index (THI) (Table 2) using the following formula:

0.8×T+RH×(T−14.4)+46.4

**Table 2:** Trailer temperature and humidity index within trailers used to transport two groups of 7–8 mo old beef calves (group 1 and 2) for a 36 h and 8 h period.

	Temperature Humidity Index (THI)		
	Minimum	Maximum	Average
*Group 1*			
36h	18.9	90.2	53.3
4h	29.8	88.0	53.6
*Group 2*			
36h	18.9	89.4	50.4
4h	39.02	77.9	49.8

### Sample collection

Weight and rectal temperature were recorded from all experimental calves (*n* = 320) and a subset of 12 calves/treatment (3 calves/pen) were sampled for physiological and behavioural indicators of welfare. Calves were sampled prior to (LO1) and after (UN1) the 36 h transport, and prior to (LO2) and after (UN2) the additional 4 h transport. In addition, calves were sampled on d 1, 2, 3, 5, 14 and 28 after UN2.

### Behavioural assessments

#### Standing and lying

Standing and lying behaviour of a subset of 12 calves/treatment was recorded with accelerometers (Hobo pendant G, Onset Computer Corporation, Bourne, MA, USA) attached to the right hind leg of the calves using Vet Wrap (Professional Preference, Calgary, AB, Canada). At LO1, accelerometers were placed, each in a vertical position on the hind right leg of the respective calf with the X-axis pointing up towards the backbone of the calf, and set to record data at 1-min intervals. Data from the days when the accelerometers were placed (d -2 and/or -1) and removed (d 5) from the calves were excluded from the analysis due to incomplete data. Standing and lying percentage per day, and standing and lying mean bout duration per day were summarized by day for further analysis.

#### Calf attitude and gait score

Calf attitude and gait were assessed after UN1 and UN2 as described previously by Meléndez et al. [7]. An experienced observer assessed calves after exiting the squeeze chute while walking down an alley outside of the handling facilities.

Attitude was evaluated using a 4-point scale [20]. Normal, bright, and alert cattle that hold their head up and readily move away from the observer receive a score of 0. Cattle that are slightly depressed but respond quickly to the observer and appear normal receive a score of 1. Cattle with moderate depression, that stand with their head down, ears drooped, with an abdomen that lacks fill and may appear floppy, and move away slowly from observer receive a score of 2. Cattle with severe depression, that stand with the head down, are very reluctant to move, and have very noticeable gauntness of abdomen receive a score of 3.

Gait score was evaluated using a 5-point scale [21]. Cattle that walk normallly, with no apparent lameness or change in gait are characterized as “walking normally” and receive a score of 0. Cattle that walk easily and readily, the line of the backbone is normal and bear full weight on all four limbs, but have an observable gait alteration are characterized as “mildly lame” and receive a score of 1. Cattle that are reluctant to walk, do so with a short weight-bearing phase of stride, rest the affected limb when standing, and have increased periods of recumbency are characterized as “moderately lame” and receive a score of 2. Cattle that lie down most of time, are reluctant to stand, refuse to walk without stimulus, do not bear weight on the affected limb therefore “hops” when moving, does not use limb when standing and have an arched backbone with caudoventral tip to the pelvis are characterized as “severely lame” and receive a score of 3. Cattle that are recumbent, unable to rise, and where euthanasia is often indicated are characterized as “non-ambulatory” and receive a score of 4.

#### Dry matter intake and feeding behaviour

Dry matter intake (DMI; kg/d/h), was determined by pen feed refusals recorded daily for d 0, 1 and 2 and 3 and weekly (week 1, 2, and 3) until d 28 after transport for 24 pens. Feed samples were collected on feed refusal days to determine feed dry matter intake (DMI).

Individual feeding behaviour was monitored in the remaining 8 pens using the GrowSafe feed bunk monitoring system (GrowSafe Systems, Airdrie, AB, Canada) as previously described by Melendez et al [7]. Calves were fitted with radio frequency ear tags (RFID, Allflex Livestock Intelligence, St-Hyacinthe, QC, Canada) and each pen was equipped with two tubs which recorded individual feed intake during the study period. Feeding data was used to calculate meal size (kg/meal/day), meal duration (min/meal/day), meal frequency (min/meal/day), feeding intake (kg/day/day), feeding duration (min/day) and feeding rate (g/min/day). A meal criterion of 300 s was selected based on previous studies in cattle [22, 23]. Feeding behaviour was evaluated for 1 pen per treatment (10 animals per treatment) for group 1.

#### Flight speed

The velocity at which animals exited the chute was collected at LO1, UN1, LO2, UN2, 1, 2, 3, 5, 14, and 28 d. The time it took an animal to travel a predetermined distance (2 m) was electronically recorded using two sets of light beams as described previously by Burrow et al. [24]. Flight speed of each sampling point was added as a covariate to the model as a negative relationship has been reported previously between flight speed, stress responses and weight gain [25, 26].

### Physiological assessments

#### Weight and rectal temperature

Calves were weighed while standing in a hydraulic squeeze chute (Cattlelac Cattle, Reg Cox Feedmixers Ltd, Lethbridge, AB, Canada) equipped with a weigh scale. Average daily gain (ADG) was calculated for the first week after transport by subtracting the d 5 BW from the initial (LO1) BW and dividing by the number of days on trial (A = 8 d and R = 7 d). The ADG of the second week was calculated by subtracting the d 14 BW from the d 5 BW and dividing by the number of days between sampling points (9 d). The ADG of the third and fourth week was calculated by subtracting the d 28 BW from d 14 BW and dividing by the number of days between samples (14 d). Shrink percentage was calculated for the 36 h transport (shrink 1) and the additional 4 h transport (shrink 2), using the formula: shrink = (1 - (BW after transport / BW before transport)) ×100.

#### Morbidity and mortality

Morbidity and mortality were recorded for the experimental calves over a 28-d experimental period.

#### Blood samples

Blood samples were collected from a subset of 12 calves/treatment through jugular venipuncture at LO1, UN1, LO2, UN2, 1, 2, 3, 5, 14, and 28 d after UN2. Blood samples were collected into 3, 10-mL non-additive tubes (BD vacutainer; Becton Dickinson Co., Franklin Lakes, NJ, USA) for osmolality, cortisol, non-esterified fatty acids (NEFA), haptoglobin (HP), serum amyloid-A (SAA), and creatine kinase (CK) analysis. Blood samples were also collected into a 7-mL sodium fluoride tube (BD vacutainer; Becton Dickinson Co., Franklin Lakes, NJ, USA) for L-lactate analysis, and into a 6-mL EDTA tube (BD vacutainer; Becton Dickinson Co., Franklin Lakes, NJ, USA) for complete blood cell count (CBC) analysis. Samples collected into the non-additive tubes and the sodium fluoride tube were left at room temperature for 1 h prior to centrifugation for 15 min at 2.5 × *g* at 4°C. Serum was decanted and frozen at -80°C for further analysis, with the exception of osmolality, which was analyzed immediately after centrifugation.

NEFA were collected as an indicator of fat mobilization due to feed deprivation. NEFA concentrations were quantified using a colorimetric assay (HR Series NEFA-HR (2), FUJIFILM Wako Pure Chemical Corporation, Osaka, Japan). The intra-assay CV was 3.3% and the inter-assay CV was 3.6%. L-lactate was measured as an indicator of muscle damage using an L-lactate colorimetric assay (Lactate Assay Kit, Cell Biolabs, Inc., San Diego, CA, USA) to quantify L-lactate concentrations in serum. The intra-assay CV was 3.7% and the inter-assay CV was 1.9%. CK concentrations were quantified as an indicator of muscle damage using a colorimetric assay (EnzyChrom™ Creatine Kinase Assay Kit, BioAssay Systems, Hayward, CA, USA). The intra-assay CV was 6.0% and the inter-assay CV was 2.3%. HP and SAA were collected as indicators of stress, inflammation, infection and trauma. HP concentrations were quantified using a colorimetric assay (Tridelta Development Ltd., Maynooth, Co, Kildare, Ireland), while SAA concentrations were quantified using an enzyme linked immunosorbent assay (Tridelta Development Ltd., Maynooth, Co, Kildare, Ireland). The haptoglobin intra-assay CV was 6.2% and the inter-assay CV was 5.2%. The SAA intra-assay CV was 5.6% and the inter-assay CV was 5.0%. Complete blood cell count (CBC) was measured as an indicator of immune function using a HemaTrueHematology Analyzer (Heska, Loveland, Co). Serum cortisol concentrations were collected as an indicator of acute stress and concentrations were quantified using an immunoassay kit (DetectX Kit, Arbor Assays, Ann Arbor, MI, USA). The intra-assay CV was 6.8% and the inter-assay CV was 11.3%. Osmolality (mOsm/kg) was collected as an indicator of dehydration and values were determined by freezing point depression (Multi-OSMETTE model 2430, Precision Systems Inc., Natick, MA, USA).

### Statistical analysis

The statistical methodology is similar to a previous study described by Melendez et al. [7]. Data were analyzed using mixed models due to the inclusion of fixed effects: conditioning, source, and time (nested in rest) and random effects: animal and pen. Time was nested in rest to account for the missing sampling point (LO2) for the 0 h treatment calves, which did not receive a rest. Data were analyzed using PROC GLIMMIX (SAS, version 9.4, SAS Inst. Inc., Cary, NC). Distributions from the exponential family (gamma, inverse Gaussian, log-normal, normal, exponential, and shifted *t*) for each variable were tested and selected based on the Bayesian information criterion (BIC). After selecting the distribution, covariance structures: compound symmetry (CS), heterogeneous compound symmetry (CSH), variance component structure (VC), first-order autoregressive (AR1), and heterogeneous first-order autoregressive (ARH1) were tested and selected based on the BIC value (S1 Table).

Covariates in the model varied depending upon the variable assessed. Group, time of day, breed, and flight speed were included as covariates in the analysis of NEFA, L-lactate, haptoglobin, SAA, cortisol, CK, osmolality, and CBC. Group and breed were included as covariates for the analysis of BW, ADG, rectal temperature, shrink 1 and shrink 2. Feed intake and breed were included as covariates for the analysis of a subset of animals for shrink 2. Breed was included as a covariate for the analysis of GrowSafe data and group was included as a covariate for the analysis of DMI and accelerometer data. GrowSafe and accelerometer data collected on d 0 were adjusted to the proportion of time animals were in the pen, as this varied between the 0 h and 8 h groups. Results are reported as least squares-means (*μ*) including the upper (u) and lower (l) limits at a 95% confidence. SAS PROC GLIMMIX iterated 1000 times at multiple levels of iterations (MAXOPT = 1000; NLOPTIONS MAXITER = 1000). Bonferroni’s correction for multiple comparisons was used.

Statistical significance was *p* ≤ 0.05. In some cases, the *F*-test indicated the statistical significance of an interaction but there was no statistically significant difference between comparisons of interest. In this case interactions are not discussed. Reported differences were limited to comparisons of interest, such as differences between conditioning (C and N), source (A and R) and rest stop (0 h and 8 h). For example, comparisons of interest included comparisons between treatments with the same source and rest effect, but differing conditioning effect (e.g. C-R-0h vs N-R-0h); the same conditioning and rest effect, but differing source effect (e.g. C-R-0h vs C-A-0h), or the same conditioning and source effect, but differing rest effect (e.g. C-R-0h vs C-R-8h) at a particular sampling point.

Data from 3 accelerometers (from the corresponding treatments C-A-8h, C-R-0h and N-A-0h) were excluded from the analysis due to technical issues and data from the 8h group was not collected on d 3 due to a severe snowstorm.

## Results and discussion

### Standing and lying

**Fig 1:**
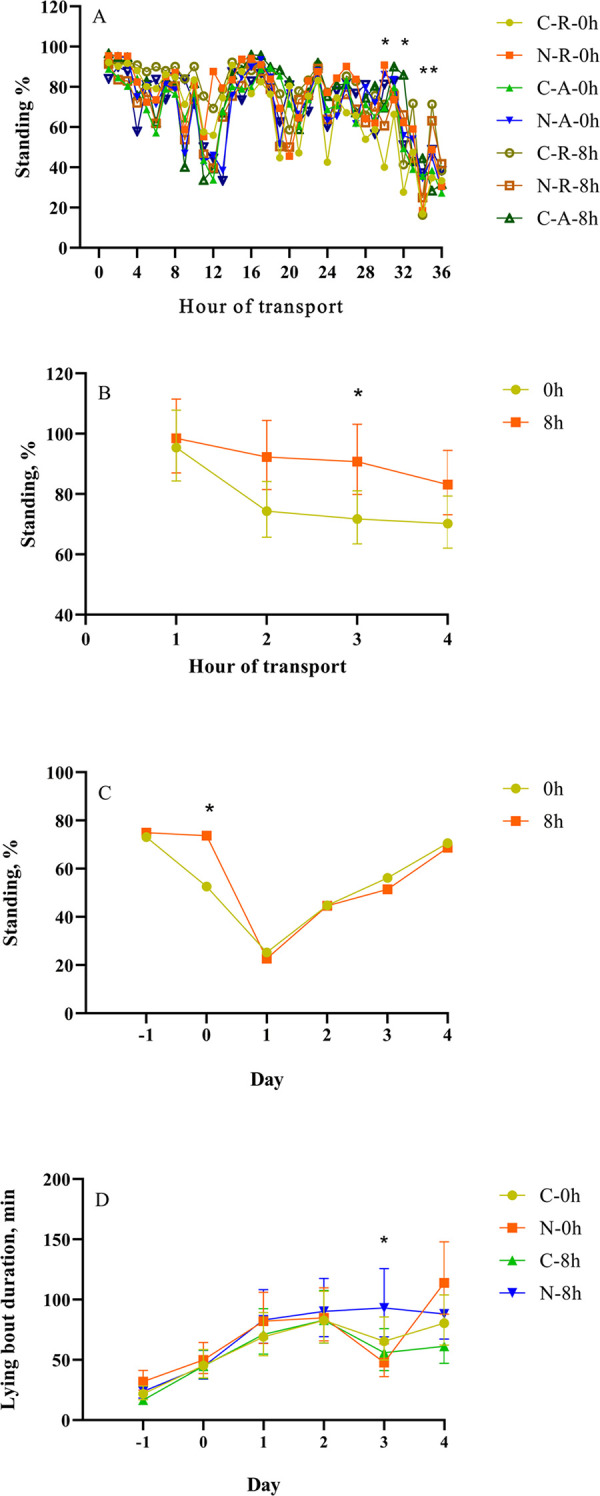
Least squares means (± upper and lower limits at 95% confidence) of standing duration (% of time) during the (A) 36 h transport, (B) additional 4 h transport, (C) first 4 days after transport and (D) lying bout duration of conditioned (C) and non-conditioned (N), auction market (A) and ranch direct (R) calves rested for 0 (0 h) or 8 (8 h) h.

The three-way interaction of conditioning × source × rest (nested in time) effect (*p* = 0.01) was observed for standing during the 36 h transport (Fig 1A; S2 Table). The mean standing percentage of the N-R-0h group *μ* = 91% (u = 124.0, l = 66.6) was greater than the C-R-0h group *μ* = 40% (u = 55.4, l = 29.0) at hour 30 of transport. The mean standing percentage of the N-R-0h group *μ* = 62% (u = 84.0, l = 46.2) was greater than the C-R-0h group *μ* = 28% (u = 37.3, l = 20.5) at hour 32 of transport. The mean standing percentage of the C-A-8h group *μ* = 44% (u = 63.4, l = 31.1) was greater than the C-R-8h group *μ* = 16% (u = 22.2, l = 11.9) at 34 h of transport, while the mean standing percentage of the C-R-8h group *μ* = 71% (u = 100.3, l = 50.8) was greater than the C-A-8h group *μ* = 28% (u = 39.1, l = 20.6) at hour 35 of transport. Rest (nested in time) affected (*p* < 0.01) standing: the mean standing percentage where the 8h rested group was greater (*p* = 0.04) standing % than the 0 h unrested group at hour 3 during the additional 4 h transport (Fig 1B).

These results were surprising because we did not expect animals to lie down during transportation, as cattle have been reported to avoid lying down when the truck is in motion [27]. Loading density may have contributed to calves lying down during the journey as the recommend loading density for 250 kg feeder calves is close to 0.70 m^2^/animal [28]. In the present study loading density ranged between 0.71 to 1.63 m^2^/animal, which may have been more conducive for calves to lie down in compartments with lower loading densities such as the nose, the back and the doghouse. Nevertheless, in the present study, treatments were equally distributed by compartments. Differences in loading densities between compartments have been reported in calves transported in Canada and the USA, where greater densities than recommended were observed in the deck and the belly compartment, while densities lower than recommended were observed in the nose, back and doghouse compartments [29]. Therefore, it was hypothesized that greater space allowance encourages animals to lie down, as animals at low and medium stocking densities change footing frequently to maintain balance, compared to animals transported under conditions of high stocking densities [30]. Treatments differences were observed towards the end of the 36 h transport. Results observed between the N and the C group were surprising as we expected the N group to lie down more due to fatigue because they were handled more prior to the 36 h transport than the C group, but the opposite was true. Differences observed between the A and R calves at 34 h could also be due to fatigue, as R calves were weaned and handled twice immediately prior to the 36 h transport than A calves which spent 24 h at the auction market prior to the 36 h transport. The difference observed at 35 h could be due to differences in behaviour observed the hour prior. The 8 h calves were characterized by numerically greater standing percentage than 0 h calves during the additional 4 h transport, however differences were only seen after 3 h of transport likely due to the lack of rest that 0 h calves experienced causing them to be more fatigued than the 8 h group.

A rest (nested in time) effect (*p* < 0.01) was observed for standing percentage after transport. The 8 h calves stood more (*p* < 0.01) than 0 h calves on d 0 (Fig 1C). This may be explained by the fact that 8h calves spent more time eating, while 0 h calves preferred to lie down due to a lack of rest, or that 8 h calves were more unsettled due to handling and transport than the 0 h calves. Differences could also be attributed to variability in the diurnal behaviour of the calves because the 0 h calves were loaded at 0900 h while the 8 h calves were loaded at 1800 h for the last 4-h journey. For example, the 0 h calves were transported in the morning, when calves are usually standing during feeding, while the 8 h calves were transported in the evening, when calves have been reported to spend more time lying [31]. These results are contrary to a previous study reporting calves not receiving a rest were characterized by greater standing durations than those that were rested [6], and another study that reported no differences in standing behaviour between conditioned calves transported for 36 h and rested for 0, 4, 8 and 12 h [7]. Interestingly, no differences were observed between treatments on d 1, 2, 3 and 4 after transport. A possible explanation for this is that calves laid down during the 36 and additional 4 h transport in the previous study [7] and present study, as loading densities were similar in both studies (0.62 to 1.18 m^2^/animal; unpublished data from previous study).

A conditioning and a source effect (*p* < 0.01) were also observed for standing percentage. Mean standing percentage of the N calves was greater than the C calves, and mean standing percentage of the R calves was greater than the A calves (*p* < 0.01). The N calves *μ* = 45% (u = 47.6, l = 43.0) may have stood more than C calves *μ* = 41%(u = 43.1, l = 39.2) because newly weaned calves typically walk more after weaning, in an attempt to reunite with their dam [32]. The R calves *μ* = 44% (u = 47.2, l = 42.6) likely were characterized by greater standing percentages than A calves *μ* = 41% (u = 43.4, l = 0.175) because they may have been unsettled following weaning, transportation, and sampling (LO1) prior to the 36 h transport, while the A calves had the opportunity to rest for 24 h after sampling (LO1) and prior to the 36 h transport. Contrary to our results, we expected R calves to stand less due to greater fatigue than the A calves that were rested before the 36 h journey. Although differences in standing percentage were statistically significant, they were relatively small (< 4%) and may lack biological relevance. A limitation to our approach is that ‘true’ auction market calves would have been sourced from different ranches and could have displayed different behaviours compared to calves in the present study.

A conditioning × rest (nested in time) effect (*p* = 0.04) was observed for mean lying bout duration. The N-0h calves were characterized by lower (*p* < 0.05) mean lying bout durations than N-8h calves on d 3 (Fig 1D). A possible explanation for the observed differences may be that N-8h calves were more settled than N-0h calves due to the rest stop they received in transit. Nevertheless, we would have expected to see similar differences between the C-8h and the C-0h calves, and similar differences on the days prior to d 3. The shorter bout durations observed on d 3 may have been caused by a severe snowstorm that took place on that day which affected Group 1 calves only. Cattle have been previously reported to stand during snowstorms [33] and avoid lying down in cold conditions unless provided with bedding which allows cattle to reduce body surface exposure [34]. Therefore, a decrease in lying behaviour was expected for all treatments, however, the N-8h calves lying bout duration remained similar to the previous day. These results are contrary to a prior study where no differences were observed for lying bout duration between calves rested for 4, 8 and 12 h and calves that did not receive a rest [7].

### Attitude score

A rest (nested in time) effect (*p* < 0.01) was observed for attitude score, where 8 h calves were characterized by a greater mean attitude score at UN2 *μ* = 0.30 (u = 0.420, l = 0.17) than UN1 *μ* = 0.07 (u = 0.185, l = -0.03). We would have expected 8 h calves to exhibit signs of fatigue after the 36 h transport, rather than after the additional 4 h transport because the transport duration was much shorter and calves were given the opportunity to rest. A conditioning × source (*p* < 0.05) effect was observed for attitude score, where N-R calves *μ* = 0.3 (u = 0.41, l = 0.16) were characterized by a greater attitude score than C-R calves *μ*_*1*_ = 0.1 (u = 0.21, l = -0.05). These results were expected because C calves are more likely to withstand transportation related stress than N calves [35]. Nevertheless, differences observed between treatments were very small (< 0.3) and may lack biological relevance. These results are contrary to a previous study where the majority (97%) of calves transported for the same length of time as the current study were given a score of 0 (normal) for both lameness and attitude [7]. Calves in the previous [7] and present study were evaluated by the same observer so differences between studies cannot be explained by inter-observer differences. Lack of treatment differences in lameness and attitude scores in the previous study may have been the result of calves being conditioned and transported fewer times than calves in the present study.

### DMI and feeding behaviour

**Fig 2:**
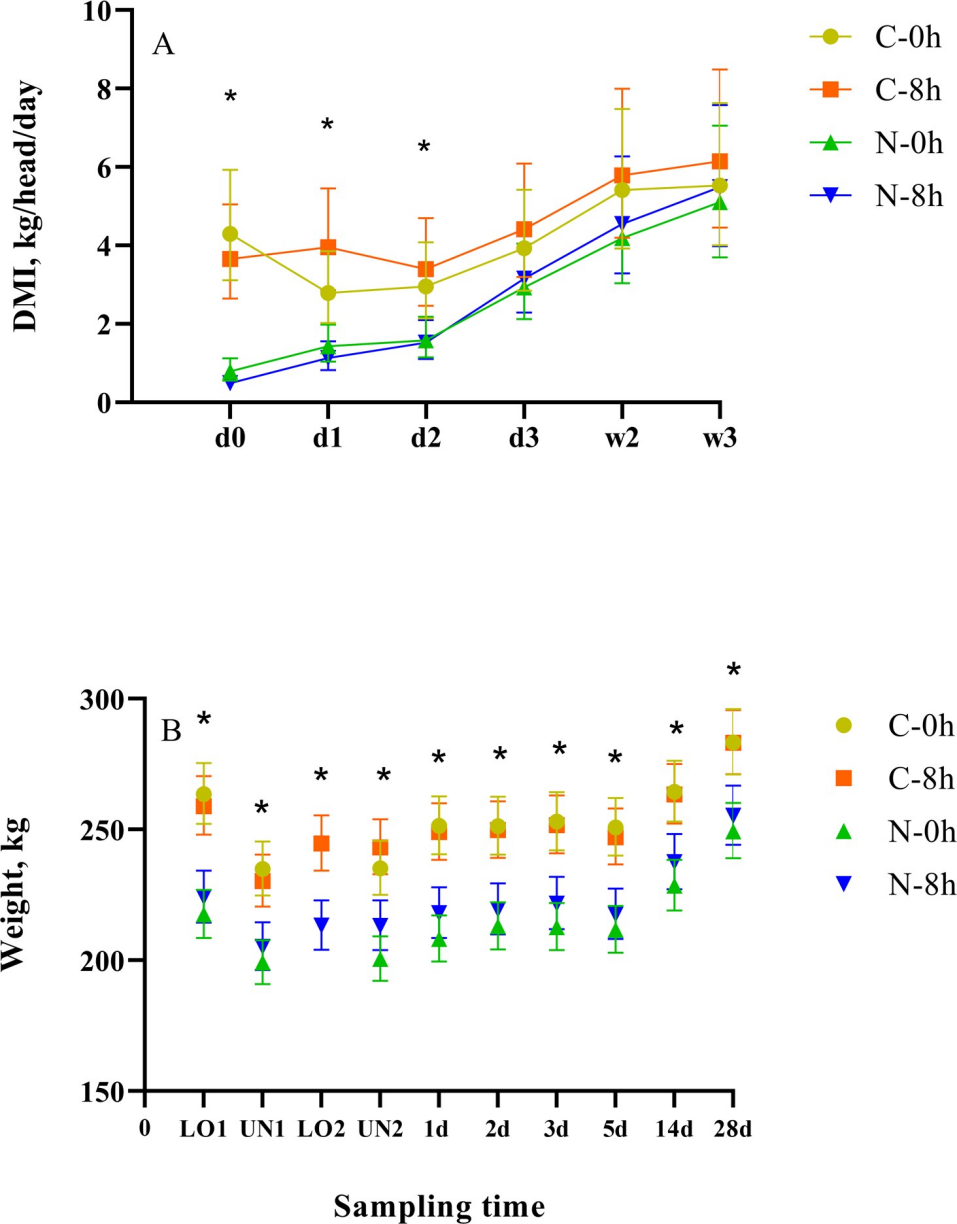
Least squares means (± upper and lower limits) of (A) DMI and (B) body weight of conditioned (C) and non-conditioned (N), auction market (A) and ranch direct (R) calves rested for 0 (0 h) or 8 (8 h) h.

A conditioning × rest (nested in time) effect (*p* < 0.01) was observed for the DMI. Both C-0h and C-8h calves were characterized by greater (*p* ≤ 0.05) DMI than N-0h and N-8h calves on d 0 and 1 (Fig 2A). The C-8h calves characterized by greater (*p* < 0.01) DMI than N-8h calves on d 2. These results are in agreement with the feeding behaviour data where overall, the C calves were characterized by greater mean feeding durations, intake, feeding rate, meal duration and meal size than N calves on d 0, 1, and 2. Results were inconsistent between treatments from d 3 to 28 (S1 File). This is likely due to C calves being adapted to the feed and the feed bunk.

### Body weight

A conditioning × rest (nested in time) effect (*p* < 0.01) was observed for mean BW, where C-0h was characterized by greater BW than the N-0h calves and the C-8h calves were characterized by greater BW than the N-8h calves BW at each sampling point (*p* < 0.01) (Fig 2B). This was expected because conditioned calves were adapted to a grain diet and the feed bunk 20 to 21 days before the start of the trial. Similar results were reported by Bailey et al. [36] where a linear relationship was observed between BW and days since weaning, and greater BW was observed in weaned than newly weaned calves prior to transport and during receiving.

### Shrink

A conditioning effect (*p* < 0.01) was observed for shrink after 36 h of transport, where C calves *μ* = 10% (u = 11.9, l = 9.5) characterized by greater shrink than N calves *μ* = 8% (u = 9.2, l = 7.4). Conditioned calves likely had more gut fill to lose during transport than N calves that had been recently weaned and transported from their ranch of origin. These results are contrary to a previous study were greater shrink was observed in newly weaned calves compared to calves weaned 15, 30, 45, and 60 days prior to transport [36]. Differences between studies could be due to differences in transportation time as in the previous study calves were transported for 4 h from the ranch of origin to an auction market where they stayed for 14 h, and were then transported for 1 h to a feedlot. This is substantially less transport time prior to and after the rest stop than in the present study. Similar results have been reported in previous studies, where greater shrink was observed when weaning was done closer to the time of transportation [35, 37].

No differences (*p* > 0.10) were observed between treatment groups for shrink after the additional 4 h transport. Data from a subset of 1 pen (10 calves/ treatment) housed in feed monitoring pens were analyzed to account for feed intake during the rest period. These results are similar to a previous study were no differences were observed in shrink after the additional 4 h of transportation between conditioned calves that received 0 h and 8 h of rest [7]. There are conflicting results in the literature on shrink, for example, Marti et al. [6] found no differences in shrink after 15 h of transport, 0, 5, 10 or 15 h of rest, and an additional 5 h of transport in newly weaned calves, and Cooke et al. [5] found differences in shrink between preconditioned calves that received 2 h of rest after 430 km of transport, and calves that did not receive a rest.

### ADG

Conditioning and a source effects (*p* < 0.01) were observed for ADG during the first week after transportation, where N *μ* = -0.2 kg (u = -0.10, l = -0.25) were characterized by greater ADG than C *μ* = -0.3 kg (u = -0.27, l = -0.42), and R *μ* = -0.2 kg (u = -0.11, l = -0.27) calves were characterized by greater ADG than A *μ* = -0.3 kg (u = -0.25, l = -0.40) calves. Greater ADG in N calves was likely due to a weight gain caused by a diet change, from an all forage low energy diet to a higher energy backgrounding (85% forage: 12% grain) diet [38]. Similar results have been reported where newly weaned calves were characterized by greater ADG compared to preconditioned calves [38], and conditioned calves after a long-haul transport [34] during the first 30 d after entry to the feedlot. Contrary to our results, no weight gain differences were observed between 20 d conditioned and newly weaned calves during the first 30 d in the feedlot, which the authors attributed to the short (< 45 d) conditioning period [39]. Greater ADG observed in R calves could be due to A calves being exposed to a new environment (auction market) and extra handling (ring simulation) prior to the 36 h transport, which could have caused stress and reduced feed consumption, however no feed intake differences were observed between R and A calves.

A source × conditioning effect (*p* = 0.03) and a rest effect (*p* <0.01) were observed for ADG during the second week. The C-A *μ* = 0.5 kg (u = 0.56, l = 0.42) and N-R *μ* = 0.5 kg (u = 0.59, l = 0.46) calves were characterized by greater (*p* <0.01) ADG than the C-R calves *μ* = 0.4 kg (u = -0.42, l = 0.29 kg). The 8 h calves *μ* = 0.5 kg (u = 0.58, l = 0.47) were characterized by greater (*p* <0.01) ADG than the 0 h calves *μ* = 0.4 kg (u = 0.50, l = 0.39). Differences between N and C calves could be due to the observed weight gain in N calves once they started to eat a higher energy diet containing some grain, similar to the results observed in week 1. Differences observed between rest treatments could be because of the opportunity (or lack of) to rest and eat for 8 h prior to the additional 4 h transport. Calves that did not receive a rest may have been more fatigued and consequently preferred lying down rather than eating after the additional 4h transport, which could have affected ADG, however, no differences were observed in feed intake.

A conditioning × rest effect (*p* < 0.01) was observed for ADG between d 14 and d 28, where C-8h *μ* = 0.6 kg (u = 0.69, l = 0.51) and N-0h calves *μ* = 0.6 kg (u = 0.67, l = 0.51) were characterized by greater ADG than N-8h calves *μ* = 0.5 kg (u = 0.58, l = 0.41). Contrary to the first two weeks after transport, C were characterized by greater ADG than N calves and 0 h were characterized by greater ADG than the 8 h calves. This could be because during the first 2 weeks after transportation the rate of weight gain in N calves would be greater due to the change in energy (added grain) in their diet. The combined stress of transport and lack of rest may have affected the ADG of 0 h calves during the first two weeks after transport, while 8 h calves benefited from the rest which resulted in greater ADG during the first 2 weeks after transport. In a previous study, heifers were reported to take 2 weeks to reach a normal DMI after a period of feed restriction [40]. The authors suggested that this was likely due to a compromised reticulo-rumen absorptive function and total tract barrier function. Greater ADG observed for 0 h calves on weeks 2–4 could be a reflection of the transportation stress which occurred earlier than in 8 h calves. This is in agreement with a study on transported calves that found 0 or 5 h rested calves were characterized by greater ADG than those receiving 10 or 15 h of rest [6]. Nevertheless, the results are contrary to a previous studies that reported no differences in ADG for conditioned calves transported 36 h and rested for 0, 4, 8 or 12 h over a 28 d period post-transport [7], or preconditioned calves with or without rest [5]. Differences in ADG between our study and previous studies could be because previous studies assessed ADG for a 30 d period instead of weekly, and had different management procedures (conditioned vs newly weaned) as well as transport and rest time.

### Morbidity and mortality

During the experimental period mortality was 0% and morbidity was 5.9%. A total of 19 calves were treated; 18 due to fever (3 C-R-0h calves, 4 C-R-8h calves, 4 C-A-0h calves, 1 C-A-8h calf, 4 N-R-8h calves and 2 N-A-8h calves), and one due to bloat (C-R-0h). Similar to a previous study [35], low morbidity in the present study was likely due to calves being purchased from a single source and not being comingled with other animals, which reduces the exposure to pathogens. This is in agreement with previous studies that have observed that comingled calves purchased from multiple sources have an increased risk of BRD [41–43].

### NEFA

**Fig 3:**
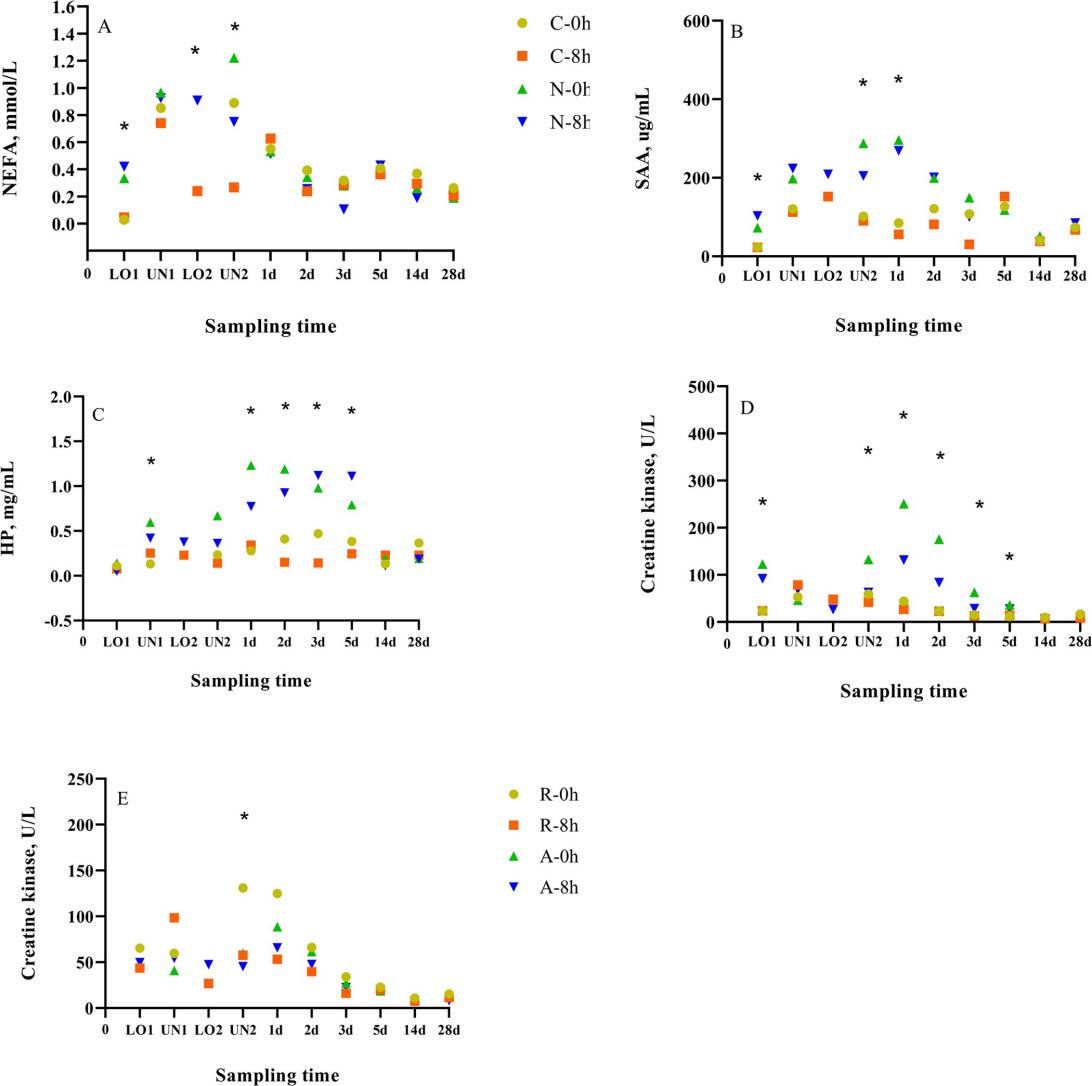
Least squares means of (A) NEFA, (B) SAA, (C) HP and (D, E) CK concentrations of conditioned (C) and non-conditioned (N), auction market (A) and ranch direct (R) calves rested for 0 (0 h) or 8 (8 h) h.

A conditioning × rest (nested in time) effect (*p* < 0.01) was observed for mean NEFA concentrations. At LO1, N-0h were characterized by greater mean NEFA concentrations than C-0h calves, and N-8h calves were characterized by greater mean NEFA concentrations than C-8h calves (*p* < 0.01) (Fig 3A; S3 Table). Increased serum NEFA concentrations are associated with food deprivation as the body mobilizes fat to use as an energy source [44]. Greater NEFA concentrations observed in N calves at LO1 were likely due to the combined effect of weaning and transport the calves underwent prior to being loaded. Weaning is a routine husbandry procedure which involves the physical separation of a calf from its dam in order for the dam to maintain or improve depleted body condition for the next pregnancy and lactation cycle [45]. It is well documented that weaning is stressful and has been shown to increase plasma cortisol [46], norepinephrine [47], and acute phase protein [48] concentrations as well as reduce immune function [49], and lying behaviour [32, 50]. Stress activates the sympathetic and inhibits the parasympathetic nervous system which is associated with activities such as resting and eating [51]. The combined effect of eliminating the opportunity for calves to suckle as well as reducing their motivation to graze [50] contributes to an overall reduction in energy intake (feed deprivation) which partially explains the increased NEFA concentrations observed in N compared to C calves. Another explanation for the greater NEFA concentrations in weaned (N) compared to C calves at LO1 is that N calves were transported for 1 h without food or water from the ranch to the research feedlot which meant they were feed (and water) deprived for at least 1 h longer, further contributing to the need to mobilize energy reserves.

No differences (*p* > 0.10) were observed between A and the R calves at LO1. These results were expected because A calves had access to feed and water at the auction market and so would not be more energy depleted than calves transported directly from the ranch. Although the provision of feed is a common practice at the auction market used in this study it is not a common practice at most other auctions within Canada as typically only water is provided. We speculate that under these typical conditions (no feed provided) NEFA concentrations could be greater in A compared to R calves.

At LO2, N-8h calves were characterized by greater (*p* < 0.01) mean NEFA concentrations than C-8h calves. These differences were likely due to N calves not eating or eating less during the rest stop. To test this theory, data from a subset of 10 animals per treatment were summarized by hour and analyzed to assess feeding behaviour during the 8 h rest period. No treatment differences were observed for feeding time (*p* > 0.10), meaning that all calves spent an equal amount of time at the feed bunk, however a conditioning × rest (nested in time) effect (*p* = 0.03) was observed for feed intake, where C-8h calves *μ =* 4.1, 1.9, 1.9, 1.5 kg/h (u = 3.31, 1 = 1.76) were characterized by greater (*p* ≤ 0.02) feed intake than N-8h calves *μ =* 1.0, 0.6, 0.9, 0.6 kg/h (u = 1.19, 1 = 0.58) at hour 1, 2, 3, and 5 of the rest period. Although no differences were observed for feed intake at hours 4, 6, 7, and 8 the C-8h calves *μ =* 1.4, 1.4, 1.8, 2.0 kg/h (u = 2.22, 1 = 1.20) were characterized by numerically greater feed intake than N-8h calves *μ =* 0.8, 0.8, 1.3, 1.4 kg/h (u = 1.45, 1 = 0.76). These results indicate that N calves were eating during the rest stop, however, the amount of feed consumed was lower than in C calves, possibly due to a lack of feed and feed bunk adaptation.

At UN2, N-8h were characterized by greater (*p* < 0.01) mean NEFA concentrations than C-8h calves. This is similar to the results observed for LO2, where N calves were characterized by lower feed intake likely due to a lack of adaptation to the feed and the feed bunk. In contrast, no treatment differences (*p* > 0.10) were observed between N-0h and C-0h calves, likely because calves did not receive a rest and therefore had no opportunity to eat.

The C-0h calves were characterized by greater (*p* = 0.01) mean NEFA concentrations than C-8h calves at UN2 which was expected because C-0h calves did not have the opportunity to eat prior to the 4 h transport. These results are in agreement with a previous study, where conditioned calves that received no rest (0h) were characterized by greater mean NEFA concentrations at UN2 than calves that received 8 h of rest after 36 h of transport [7]. We would have expected to see similar results between N-0h and N-8h calves at UN2. Although there were no differences (*p* > 0.10) between the groups, N-0h calves did have numerically greater NEFA concentrations than N-8h calves. One explanation is that N-8h calves may not have consumed enough feed during the rest period for NEFA concentrations to be different from the N-0h calves. Even though the amount of feed consumed by N calves was lower than C calves, we would have expected to see a difference between N-0h and N-8h calves, as NEFA concentrations have been reported to decrease rapidly when animals are provided feed and water [52].

### SAA and HP

A conditioning × rest (nested in time) effect (*p* < 0.01) was observed for mean SAA concentrations. The N-0h calves were characterized by greater mean SAA concentrations than C-0h calves, and N-8h calves were characterized by greater mean SAA concentrations than C-8h calves, at LO1 and on d 1 (*p* ≤ 0.01) (Fig 3B). The N-0h calves were also characterized by greater (*p* = 0.02) mean SAA concentrations than C-0h calves at UN2. A conditioning × rest (nested in time) effect (*p* < 0.01) was observed for mean HP concentrations. The N-0h calves were characterized by greater (*p* < 0.01) mean HP concentrations than C-0h calves at UN1, and on d 1 and 2 (Fig 3C). The N-8h calves were characterized by greater (*p* < 0.01) mean HP concentrations than C-8h calves on d 2, 3, and 6.

The acute phase response (APR) has been reported to peak 2 to 3 d after a stimulus that produces inflammation, tissue damage, infection or trauma [53]. This is in agreement with the differences observed in SAA concentrations between N and C calves at UN2 and on d 1, which corresponds to d 2 and 3 after weaning for the N calves. Similar to our findings, HP and SAA concentrations have been reported to increase 3 to 5 d post-weaning in 6-week-old Holstein calves [54]. Nevertheless, the effect of weaning on the APR response is inconsistent in the literature. For example, weaning did not affect SAA concentrations, while greater HP concentrations were observed on d 2 and 7 after weaning compared to baseline levels in 8-month-old calves [55]. In contrast, an association was observed between weaning and fibrinogen, while no relationship was observed between weaning and HP in 7-month-old calves [47]. Inconsistencies in the APR response in the previous studies could be due to the use of heifers and steers, which are known to have different temperaments [56] and therefore can react differently to stressful situations.

In the present study, HP and SAA values were greater than the normal range (HP: < 0.1 g/L and SAA 1.3 mg/L) for cattle [57] prior to transportation, and an increase was observed for both SAA and HP concentrations after transport. Contrary to our findings, SAA concentrations have been reported to be greater, while HP concentrations have been reported to be lower in transported than non-transported calves [58]. Differences in SAA concentrations at LO1 were unexpected, and may be a result of the combined stress associated with weaning, transportation and handling, as the APR has been suggested to be activated through the production of corticosteroids [59]. Nevertheless, no differences (*p* > 0.10) were observed in cortisol concentrations at LO1 or throughout the study. In addition, we did not expect C calves to have SAA and HP concentrations above the normal range at LO1. Conditioned calves may have been stressed when mixed with conditioned calves from other pens and non-conditioned calves prior to LO1 sampling, however, we would not expect to see an increase in concentrations shortly after a stressor as indicated earlier.

Differences have been reported between SAA and HP responses to the same stimuli [55, 57]. Differences in SAA concentrations for the 0 h and 8 h groups were observed at the same sampling points (LO1, UN2 and d1), whereas differences in HP were observed earlier in the 0 h calves (UN1, d 1 and 2) and later (d 2, 3 and 6) in the 8 h calves. The HP response may have been prolonged in the 8 h calves due to a delay in the time of arrival of the calves to their final destination. High variability (0.95 to 581 μg/mL) was observed for SAA concentrations, so caution should be taken when interpreting these results.

### Creatine kinase

A conditioning × rest (nested in time) effect (*p* < 0.01) was observed for mean CK concentrations. At LO1, N-0h were characterized by greater mean CK concentrations than C-0h calves, and N-8h calves were characterized by greater mean CK concentrations than C-8h calves (*p* < 0.01) (Fig 3D). Creatine kinase is an enzyme involved in the production of ATP in the muscle [60], which increases after muscular activity due to a higher utilization of energy [61] and appears in the plasma as a result of muscle cell damage [62]. Creatine kinase has been previously used in transport research as an indicator of muscle fatigue, because transported cattle typically stand over the course of their journey and have to maintain their balance during that time. Concentrations of CK have been reported to increase progressively with time of transport [27]. Higher CK concentrations in N calves prior to the 36 h transport could be due to greater physical activity associated with the gathering of the cow-calf pairs at the ranch and weaning which can increase walking behaviour for calves in an attempt to reunite with the dams [32, 50]. In addition, calves were transported for 1 h from the ranch of origin to the research feedlot, while C calves was housed at the research feedlot. The CK results are similar to those observed for NEFA and SAA.

At UN2, d 1, 2, 3 and 6, N-0h calves were characterized by greater (*p* < 0.01) mean CK concentrations than C-0h calves. On d 1 and 2, N-8h calves were characterized by greater (*p* < 0.01) mean CK concentrations than C-8h calves. These results suggest that N calves had greater muscle fatigue than C calves, and that differences were observed for a longer period of time in N-0h compared to N-8h calves, likely due to recovery of the 8h calves during the rest period. This is contrary to the HP findings where differences in 8h calves were observed at a later time point than the 0 h calves. Differences between these parameters may be because CK is associated with muscle fatigue, while HP concentrations can increase due to stimuli that cause inflammation, infection, stress, or trauma.

A source × rest (nested in time) effect (*p* = 0.01) was observed for mean CK concentrations. At UN2, R-0h calves were characterized by greater (*p* < 0.01) mean CK concentrations than A-0h calves (Fig 3E). This is likely due to the ranch calves having greater physical activity prior to the 36 h transport as ranch calves were weaned, transported and sampled, while the auction market calves were only put through the ring prior to the 36 h transport. Greater CK and NEFA concentrations and lower muscle glycogen have been reported in bulls mixed with unfamiliar animals [63], likely due to an increase in physical activity associated with antagonistic behaviours. Nevertheless, we would have expected to see similar differences between the R-8h and the A-8h calves. Lack of differences could be because R-8h and A-8h calves received a rest prior to the additional 4 h transport, which allowed calves to eat and recover. Differences observed between R-0h and A-0h could be due to the lack of rest, as energy deficiency and muscle glycogen depletion have been associated with muscle fatigue during prolonged exercise [64].

### Osmolality and HCT

A source × conditioning × rest (nested in time) effect (*p* = 0.05) was observed for osmolality, however no differences were observed between comparisons of interest. Osmolality is a measure of the total number of dissolved solute particles in one kilogram of a solution. Plasma osmolality has been previously used as is an indicator of hydration in cattle [65, 66]. Greater osmolality would be expected in animals that have not had access to water, as solutes would be more concentrated in their plasma. Another indicator used to assess hydration is hematocrit, which is the percentage of blood volume occupied by red blood cells. Vogel et al. [66] suggested that plasma osmolality may be more sensitive to changes in cattle hydration than hematocrit (HCT) since they found that osmolality was greater in feed and water deprived slaughter cows than those that had access to feed and water. However, they did not observe any differences in HCT assessed in the same cows. In the present study, no differences were observed in osmolality or HCT between treatments of interest. These findings were unexpected, as we would have predicted that calves receiving 8 h of rest would have had lower plasma osmolality than calves not receiving a rest at the UN2 sampling point. However, these findings are similar to previous studies that observed no clinical signs of dehydration in young calves after long transport journeys [67–69]. Lack of differences between treatments could be due to a short additional 4 h transport that may not be long enough to cause a substantial change in hydration between calves rested 0 or 8 h. Similarly, no differences were observed in HCT in calves transported either 12 or 36 h and rested for either 0, 4, 8 or 12 h [7]. The authors suggested that lack of treatment differences in hematocrit could be due to water absorption from the ruminal contents [62].

### L-lactate

A source × conditioning × rest (nested in time) effect (*p* < 0.01) was observed for L-lactate concentrations. The C-R-8h calves (UN1 *μ* = 1.7 mmol/L (u = 1.91, l = 1.40), d14 *μ* = 1.0 mmol/L (u = 1.23, l = 0.71) were characterized by greater mean L-lactate concentrations than N-R-8h calves (UN1 *μ* = 1.1 mmol/L (u = 1.35, l = 0.85), d 14 *μ* = 0.6 mmol/L (u = 0.86, l = 0.35) at UN1 and on d 14 post-transport. L-lactate is an indicator of muscle damage (similar to CK), however, it can also be produced in other tissues in the body [62]. These findings were surprising, as L-lactate is an indicator of muscle fatigue related to exercise intensity and therefore we expected to observe findings similar to what was observed for CK. Contrary to NEFA, SAA, HP, and CK, lactate concentrations were greater in C than N calves at UN1 and d 14. This may be due to lactate not being as muscle specific as CK. Based on these results lactate may not be a sensitive indicator of muscle fatigue.

### Complete blood cell count

A conditioning × rest (nested in time) effect (*p* = 0.05) was observed for WBC count. The N-8h calves were characterized by greater (*p* ≤ 0.03) WBC count than C-8h calves at LO1, and on d 1 and 2 post-transport (Fig 3F). Interestingly, no differences (*p* > 0.10) were observed between N-0h and C-0h calves. WBC counts were higher in all treatments than the reported normal range (4–12 × 10^3^/μL) for beef calves [70]. Increased WBC count has been associated with stress, excitation, fear or exercise [71]. Greater WBC counts in N compared to C calves at LO1 may be explained by stress and fear as well as increased exercise associated with weaning, and transportation. These results are in agreement with our findings for NEFA, SAA, and CK.

**Fig 4:**
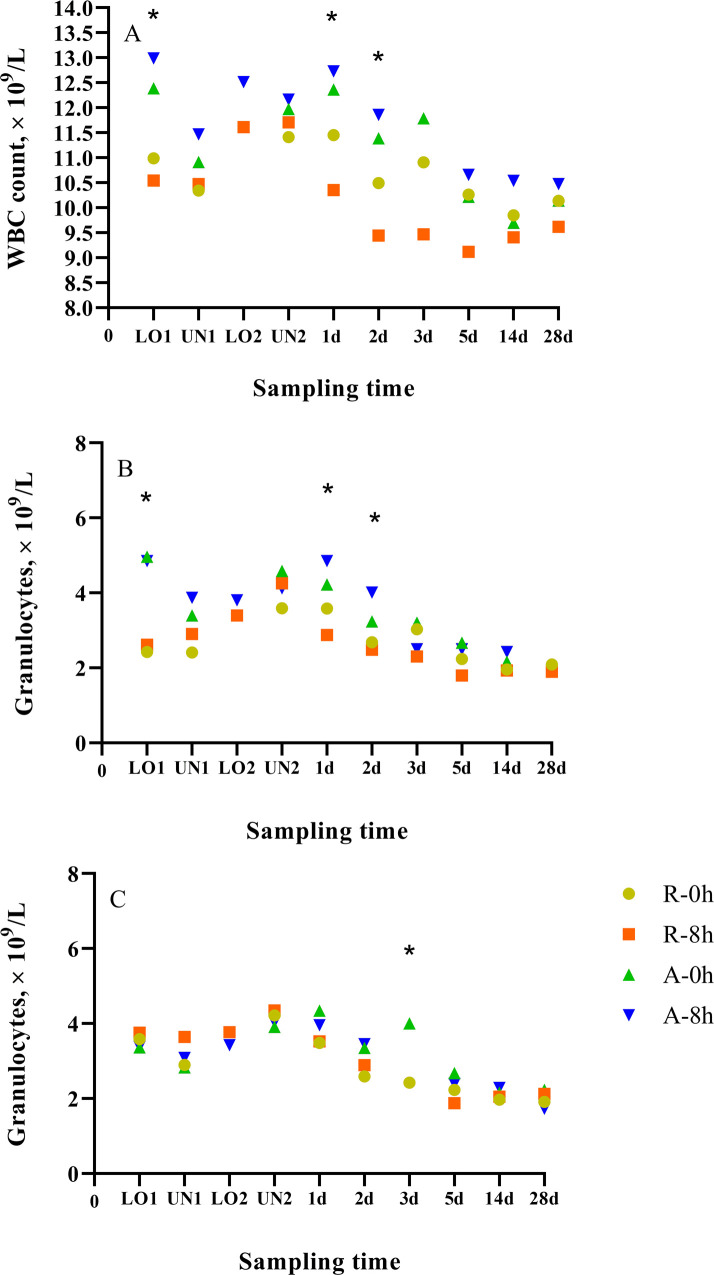
Least squares-means of (A) WBC and (B and C) granulocyte count of conditioned (C) and non-conditioned (N), auction market (A) and ranch direct (R) calves rested for 0 (0 h) or 8 (8 h) h.

A conditioning × rest (nested in time) effect (*p* < 0.01) was observed for granulocyte count. The mean granulocyte count for the C-0h calves was greater (*p* < 0.01) than the N-0h calves, and the mean granulocyte count of the C-8h calves was greater (*p* < 0.01) than N-8h calves at LO1 (Fig 4A). In addition, the N-8h calves were characterized by greater (*p* < 0.01) granulocyte counts than C-8h calves on d 1 and 2 post-transport. Similar to the results for WBC counts, mean granulocyte counts were greater in N calves than C calves except at LO1. This is contrary to what we expected as N calves were recently weaned and transported and we would have expected calves to be more stressed than C calves. A source × rest (nested in time) effect (*p* < 0.01) was observed for granulocyte counts. The A-0h calves were characterized by greater (*p* = 0.01) granulocyte counts than R-0h calves on d 3 post-transport (Fig 4B). Although statistical differences were observed between treatments, the granulocyte counts of all calves fell within the normal range (1.9–7.9 10^3^/ul) reported for beef cattle [70].

Lack of differences in physiological parameters and CBC between R and A calves in the present study could be because calves were not comingled at the auction market and were provided with feed and water. Comingling can cause stress due to mixing with unfamiliar calves, increasing the risk of disease due to exposure to pathogens [13]. Calves were not comingled in the present study due to time and labor limitations. Feed deprivation has also been reported to cause a physiological response [52]. Based on these results, exposure to auction market conditions had little effect on the physiology and behaviour of calves. Future transport studies should include cattle that have been comingled and feed deprived to assess the effect of auction market conditions (comingling and feed deprivation) on physiological and behavioural indicators of stress.

## Conclusions

Based on the observed physiological and behavioral variables, with the exception of NEFA and standing %, there were few statistically significant differences between rested and unrested calves. Few and inconsistent indicators of reduced welfare were observed between auction market and ranch direct calves, while non-conditioned calves were characterized by greater physiological and behavioural indicators of reduced welfare than conditioned calves. The lack of statistically significant differences between the experimental rest durations was attributed to cattle lying down during transport and the additional 4 h of transport is hypothesized to be insufficient for rest to affect welfare indicators. The lack of statistically significant effect due to source may be due to calves not being comingled and the accessibility of feed and water at the auction market. Based on the findings of this study: conditioning can improve the cattle welfare during transportation, auction markets should provide feed to animals as a management strategy to minimize stress, and rest did not consistently affect the welfare of beef cattle. Future studies should assess the effect of rest with a longer transportation time following the rest period, and the effect of rest on auction market feed deprived and commingled cattle.

## Supporting information

S1 Table Generalized linear mixed modelling (SAS POC GLIMMIX statements) indicating the response variable, the selected distribution, the link function, and the selected structure of the covariance matrix. (DOCX)

S2 Table Least squares-means (± upper and lower limits at 95% confidence) of production and behavioural parameters of conditioned (C) and non-conditioned (N), auction market (A) and ranch direct (R) calves rested for 0 (0 h) or 8 (8 h) h.
(DOCX)

S3 Table Least squares-means (± upper and lower limits at 95% confidence) of physiologic parameters of conditioned (C) and non-conditioned (N), auction market (A) and ranch direct (R) calves rested for 0 (0 h) or 8 (8 h) h^1^.
(DOCX)

S1 File **Detailed feeding behavior results and least squares-means of feeding behavior for conditioned (C) or non-conditioned (N), auction market (A) or ranch direct (R) calves rested for 0 (0 h) or 8 (8 h) h.** (A) Feeding rate, (B) feeding time, (C) meal frequency, (D) meal size, (E) feeding intake, and (F and G) meal duration.
(DOCX)

S1 Data (XLSX)
