# Supplementary material for: Effects of conditioning, source, and rest on indicators of stress in beef cattle transported by road
Source: PLoS One. 2021 Jan 12;16(1):e0244854. doi: 10.1371/journal.pone.0244854 (PMC7803389; doi:10.1371/journal.pone.0244854)
Supplement: S1 Table — (DOCX) [file pone.0244854.s001.docx]

S1 Table. Generalized linear mixed modelling (SAS POC GLIMMIX statements) indicating the response variable, the selected distribution, the link function, and the selected structure of the covariance matrix.

| **Experimental variable** | **Distribution** | **Link function** | **Covariance structure** |
| --- | --- | --- | --- |
| NEFA | N | Identity | ARH(1) |
| Haptoglobin | N | Identity | ARH(1) |
| SAA | GAMMA | Reciprocal Square | AR(1) |
| Serum cortisol | GAMMA | Log | VC |
| L-Lactate | N | Identity | VC |
| Creatine Kinase | LOGN | Identity | AR(1) |
| Osmolality | LOGN | Identity | VC |
| Weight | GAMMA | Log | AR(1) |
| ADG | T | Identity | - |
| Shrink 1 | LOGN | Identity | - |
| Shrink 2 | LOGN | Identity | - |
| Feeding Time | LOGN | Identity | VC |
| Feeding Intake | GAMMA | Log | VC |
| Feeding Rate | LOGN | Identity | VC |
| Meal frequency | LOGN | Identity | VC |
| Meal duration | LOGN | Identity | VC |
| Meal size | GAMMA | Log | VC |
| Flight Speed | GAMMA | Log | VC |
| DMI | GAMMA | Log | - |
| Standing bout duration | LOGN | Reciprocal Square | - |
| Lying bout duration | IG | Log | - |
| Standing percentage | IG | Log | - |
| Lying percentage | GAMMA | Log | - |
| Attitude score | T | Identity | ARH(1) |
| WBC | GAMMA | Log | AR(1) |
| Granulocytes | LOGN | Identity | CSH |
| HCT | GAMMA | Log | AR(1) |
