# Supplementary material for: Effects of conditioning, source, and rest on indicators of stress in beef cattle transported by road
Source: PLoS One. 2021 Jan 12;16(1):e0244854. doi: 10.1371/journal.pone.0244854 (PMC7803389; doi:10.1371/journal.pone.0244854)
Supplement: S2 Table — Least squares-means (± upper and lower limits at 95% confidence) of production and behavioural parameters of conditioned (C) and non-conditioned (N), auction market (A) and ranch direct (R) calves rested for 0 (0 h) or 8 (8 h) h. (DOCX) [file pone.0244854.s002.docx]

S2 Table. Least squares-means (± upper and lower limits at 95% confidence) observed values of production and behavioural parameters of conditioned (C) and non-conditioned (N), auction market (A) and ranch direct (R) calves rested for 0 (0 h) or 8 (8 h) h^1^

|  | Treatment^2^ | | | | | | | |  |  | *p*-value | | | | |
| --- | --- | --- | --- | --- | --- | --- | --- | --- | --- | --- | --- | --- | --- | --- | --- |
| *Item* | C-R-0h | N-R-0h | C-A-0h | N-A-0h | C-R-8h | N-R-8h | C-A-8h | N-A-8h | Lower | Upper | Cond | Sou | Cond×Sou×R | Time(R) | C×S×Time(R) |
| BW, kg | 257 | 250 | 210 | 219 | 254 | 248 | 212 | 232 | 223.5 | 248.6 | <0.01 | 0.21 | 0.48 | <0.01 | 0.62 |
| Shrink 1, % | 10.0 | 7.7 | 11.2 | 8.8 | 10.5 | 7.5 | 10.8 | 8.9 | 8.18 | 10.9 | <0.01 | <0.01 | 0.40 | - | - |
| Shrink 2, % | 2.3 | 2.2 | 2.3 | 2.4 | 2.3 | 2.1 | 2.3 | 2.3 | 2.07 | 2.49 | 0.07 | 0.06 | 0.92 | - | - |
| ADG, kg/day | 0.6 | 0.9 | 0.5 | 0.9 | 0.7 | 0.9 | 0.7 | 0.9 | 0.67 | 1.04 | <0.01 | 0.26 | 0.89 | - | - |
| DMI, kg/d/head | 4.8 | 2.6 | 3.6 | 2.8 | 5.0 | 2.4 | 4.2 | 3.0 | 2.83 | 4.55 | <0.01 | 0.77 | 0.89 | <0.01 | 0.15 |
| Meal size, kg/meal | 0.5 | 0.4 | 0.5 | 0.4 | 0.5 | 0.4 | 0.5 | 0.4 | 0.46 | 0.52 | <0.01 | 0.05 | 0.31 | <0.01 | <0.01 |
| Meal duration, min/meal | 12.5 | 12.7 | 12.6 | 13.1 | 12.3 | 13.5 | 11.8 | 12.1 | 11.04 | 14.41 | <0.01 | 0.06 | 0.65 | <0.01 | 0.45 |
| Meal frequency meals/day | 13.8 | 12.9 | 13.1 | 14.4 | 13.2 | 14.3 | 13.6 | 14.9 | 12.56 | 15.22 | <0.01 | 0.01 | 0.22 | <0.01 | <0.01 |
| Feed intake, kg/day | 6.9 | 6.0 | 7.2 | 5.8 | 7.1 | 6.3 | 7.1 | 6.0 | 5.90 | 7.38 | <0.01 | 0.53 | 0.90 | <0.01 | 0.14 |
| Feeding rate, g/min | 38.7 | 35.8 | 42.6 | 29.2 | 42.9 | 30.9 | 43.9 | 31.7 | 34.77 | 39.37 | <0.01 | 0.15 | <0.01 | <0.01 | <0.01 |
| Feeding time, min/day | 173 | 162 | 161 | 189 | 161 | 192 | 158 | 180 | 153.8 | 193.7 | <0.01 | 0.97 | 0.09 | <0.01 | 0.32 |
| Lying, % | 43 | 38 | 44 | 42 | 51 | 49 | 48 | 49 | 35.1 | 59.8 | 0.69 | 0.77 | 0.81 | <0.01 | 0.04 |
| Standing, % | 54 | 58 | 52 | 55 | 57 | 59 | 58 | 59 | 49.5 | 65.6 | 0.08 | 0.41 | 0.93 | <0.01 | 0.40 |
| Lying bout, min | 55 | 70 | 68 | 71 | 55 | 70 | 57 | 72 | 52.3 | 81.5 | 0.55 | 0.87 | 0.89 | <0.01 | 0.43 |
| Standing bout, min | 65 | 93 | 74 | 84 | 84 | 100 | 99 | 102 | 70.4 | 110.4 | 0.24 | 0.66 | 0.21 | <0.01 | 0.93 |
| Flight speed, m/s | 1.8 | 1.4 | 1.7 | 1.4 | 1.9 | 1.4 | 1.3 | 1.1 | 1.17 | 2.09 | <0.01 | 0.05 | 0.99 | <0.01 | 0.98 |
| Attitude score | 0.1 | 0.2 | 0.1 | 0.1 | 0.1 | 0.3 | 0.1 | 0.1 | 0.02 | 0.31 | 0.01 | 0.56 | 0.99 | <0.01 | 0.86 |

Scheffe *p*-values are presented in the table.

^1^Values in the table represent the means of body weight (BW), ADG and flight speed for LO1, UN1, LO2, UN2, 1, 2, 3, 5, 14 and 28 d; the means of shrink 1 for UN1 and shrink 2 for UN2, the means for feed refusals for d 0, 1, and 2, and week 1, 2, and 3, the means of meal size, meal duration, meal frequency, feed intake, feeding rate, feeding time for d 0 to d 28; the means for UN1 and UN2 for attitude score.

^2^ Conditioning: C: conditioned and N: non-conditioned calves. Source: R: ranch direct and A: auction market calves. Rest stop: 0 h: no rest and 8 h: 8 h of rest.
