# Supplementary material for: Effects of conditioning, source, and rest on indicators of stress in beef cattle transported by road
Source: PLoS One. 2021 Jan 12;16(1):e0244854. doi: 10.1371/journal.pone.0244854 (PMC7803389; doi:10.1371/journal.pone.0244854)
Supplement: S3 Table — Least squares-means (± upper and lower limits at 95% confidence) of physiologic parameters of conditioned (C) and non-conditioned (N), auction market (A) and ranch direct (R) calves rested for 0 (0 h) or 8 (8 h) h1. (DOCX) [file pone.0244854.s003.docx]

S3 Table. Least squares-means (± upper and lower limits at 95% confidence) of physiologic parameters of conditioned (C) and non-conditioned (N), auction market (A) and ranch direct (R) calves rested for 0 (0 h) or 8 (8 h) h^1^

|  | Treatment^2^ | | | | | | | |  |  | *p* -value | | | | |
| --- | --- | --- | --- | --- | --- | --- | --- | --- | --- | --- | --- | --- | --- | --- | --- |
| *Item* | C-R-0h | N-R-0h | C-A-0h | N-A-0h | C-R-8h | N-R-8h | C-A-8h | N-A-8h | Lower | Upper | Cond | Sou | Cond×Sou×R | Time(R) | C×S×Time(R) |
| Cortisol, ng/mL | 19 | 18 | 22 | 22 | 21 | 17 | 20 | 19 | 16.1 | 24.3 | 0.27 | 0.16 | 0.53 | <0.01 | 0.09 |
| SAA, ng/mL | 103 | 176 | 131 | 180 | 102 | 168 | 100 | 154 | 111.1 | 175.7 | <0.01 | 0.14 | 0.55 | <0.01 | 0.70 |
| HP, mg/mL | 0.2 | 0.5 | 0.4 | 0.8 | 0.1 | 0.5 | 0.3 | 0.5^b^ | 0.23 | 0.62 | <0.01 | 0.02 | 0.05 | <0.01 | 0.73 |
| L-lactate, mM | 1.1 | 0.8 | 0.8 | 0.7 | 0.8 | 0.6 | 0.7 | 0.7 | 0.79 | 1.12 | <0.01 | 0.26 | 0.19 | <0.01 | <0.01 |
| NEFA, mmol/L | 0.4 | 0.5 | 0.5 | 0.4 | 0.2 | 0.4 | 0.3 | 0.4 | 0.35 | 0.54 | 0.01 | 0.89 | 0.71 | <0.01 | 0.35 |
| Creatine kinase, U/L | 33 | 75 | 23 | 63 | 18 | 34 | 20 | 30 | 28.9 | 48.4 | <0.01 | 0.09 | 0.15 | <0.01 | 0.24 |
| Osmolality, mOsm | 295 | 300 | 296 | 300 | 296 | 299 | 296 | 297 | 295.1 | 300.3 | <0.01 | 0.69 | 0.15 | <0.01 | 0.05 |
| HCT, % | 32 | 34 | 34 | 33 | 31 | 32 | 31 | 31 | 32.0 | 33.5 | 0.79 | 0.89 | 0.20 | <0.01 | 0.71 |
| WBC, ×10^3^/µL | 10 | 10 | 11 | 11 | 10 | 11 | 9 | 12 | 10.4 | 11.5 | 0.01 | 0.20 | 0.26 | <0.01 | 0.21 |
| Granulocytes, 10^3^/µL | 2.7 | 3.3 | 2.8 | 3.7 | 2.8 | 3.2 | 2.4 | 3.4 | 2.5 | 3.7 | <0.01 | 0.34 | 0.51 | <0.01 | 0.46 |

Scheffe *P*-values are presented in the table.

^1^Values in the table represent the mean of LO1, UN1, LO2, UN2, d 1, 2, 3, 5, 14 and 28 of cortisol, serum amyloid A (SAA), haptoglobin (HP), L-lactate, non-estrified fatty acids (NEFA), creatine kinase, osmolality, hematocrit (HCT), white blood cells (WBC), and granulocytes.

^2^ Conditioning: C: conditioned and N: non-conditioned calves. Source: R: ranch direct and A: auction market calves. Rest stop: 0 h: no rest and 8 h: 8 h of rest.
