# Supplementary material for: Effects of conditioning, source, and rest on indicators of stress in beef cattle transported by road
Source: PLoS One. 2021 Jan 12;16(1):e0244854. doi: 10.1371/journal.pone.0244854 (PMC7803389; doi:10.1371/journal.pone.0244854)
Supplement: S1 File — Detailed feeding behavior results and least squares-means of feeding behavior for conditioned (C) or non-conditioned (N), auction market (A) or ranch direct (R) calves rested for 0 (0 h) or 8 (8 h) h. (A) Feeding rate, (B) feeding time, (C) meal frequency, (D) meal size, (E) feeding intake, and (F and G) meal duration. (DOCX) [file pone.0244854.s004.docx]

S1 File

Feeding behaviour

Feeding time

A source × conditioning × rest (nested in time) effect (*p* = 0.03) was observed for the feeding time. The C-A-0h and the N-A-8h group were characterized by greater (*p* < 0.01) feeding time than the N-A-0h group on d 0. The N-R-0h, the N-A-8h and the C-R-8h group were characterized by greater (*p* < 0.01) feeding time than the N-R-8h group on d 1.

Feeding intake

A conditioning × rest (nested in time) effect (*p* < 0.01) was observed for the feeding intake. The C-0h group was characterized by greater (*p* = 0.01) feeding intake than the N-0h group on d 0 and 1. The C-8h group was characterized by greater (*p* = 0.01) feeding intake than the N-8h group on d 1. The C-0h group was characterized by greater (*p* = 0.01) feeding intake than the N-0h group on d 2.

Feeding rate

A source × conditioning × rest (nested in time) effect (*p* < 0.01) was observed for the feeding rate. The C-R-0h group was characterized by greater (*p* < 0.01) feeding rate than the N-R-0h group on d 0 and 1. The N-R-8h group was characterized by greater (*p* < 0.01) feeding rate than the N-R-0h group on d 0. The C-A-0h group was characterized by greater (*p* < 0.01) feeding rate than the N-A-0h group on d 0. The N-A-8h group was characterized by greater (*p* < 0.01) feeding rate than the N-A-0h group on d 0. The C-A-8h group was characterized by greater (*p* < 0.01) feeding rate than the N-A-8h group on d 0 and 1. The C-R-8h group was characterized by greater (*p* < 0.01) feeding rate than the N-R-8h group on d 1. The C-R-8h group was characterized by greater (*p* < 0.01) feeding rate than the N-R-8h group on d 2. The N-R-0h, C-R-8h and the C-A-0h group were characterized by greater feeding rate (*p* < 0.01) than the C-R-0h group on d 3. The N-R-0h group was characterized by greater (*p* < 0.01) feeding rate than the N-A-0h and the N-R-8h group on d 3. The C-A-0h group was characterized by greater (*p* < 0.01) feeding rate than the N-A-0h group on d 3. The N-R-0h and the C-A-0h group were characterized by greater feeding rate (*p* ≤ 0.03) than the N-A-0h group on d 5. The N-R-0h and the C-A-0h group were characterized by greater feeding rate (*p* < 0.01) than the N-A-0h group on d 6. The C-A-0h group was characterized by greater feeding rate (*p* < 0.01) than the N-A-0h group on d 7, 8, 9, 10, 11, 12, 13, 14, 15. The C-A-8h group was characterized by greater feeding rate (*p* < 0.01) than the N-A-8h group on d 6, 7, 9, 11, 14, and 15. The C-R-8h group was characterized by greater feeding rate (*p* < 0.01) than the N-R-8h group on d 8, 9, 11, 12, 13, and 15. The N-R-0h group was characterized by greater feeding rate (*p* ≤ 0.05) than the N-A-0h group on d 25, 26 and 27.

Meal frequency

A source × conditioning × rest (nested in time) effect (*p* < 0.01) was observed for the meal frequency. The C-R-8h group was characterized by a greater meal frequency (*p* ≤ 0.05) than the C-A-8h group on d 1. The C-R-0h group was characterized by a greater meal frequency (*p* < 0.01) than the C-A-0h and the C-R-8h group on d 3. The N-A-0h group was characterized by a greater meal frequency (*p* < 0.01) than the C-A-0h group on d 3. The N-A-8h group was characterized by a greater meal frequency (*p* < 0.01) than the C-A-8h group on d 3. The N-R-8h group was characterized by a greater meal frequency (*p* = 0.02) than the N-A-8h on d 4. The N-A-0h group was characterized by a greater meal frequency (*p* = 0.01) than the N-R-0h group on d 5. The N-A-0h group was characterized by a greater meal frequency (*p* < 0.01) than the C-A-0h group on d 6. The N-A-8h group was characterized by a greater meal frequency (*p* < 0.01) than the N-R-8h group on d 18.

Meal duration

A source × rest (nested in time) effect (*p* < 0.01) was observed for the meal duration, where the A-0h group was characterized by a greater meal duration (*p* < 0.01) than the A-8h group on d 0.

A conditioning × rest (nested in time) effect (*p* < 0.01) was observed for the meal duration, where the C-8h group was characterized by a greater (*p* = 0.02) meal duration than the C-0h group on d 0, and the C-8h group was characterized by a greater meal duration (*p* = 0.05) than the N-8h group on d 0. The N-0h group was characterized by a greater meal duration (*p* < 0.01) than the N-8h group on d 1, and the N-8h group was characterized by a greater meal duration (*p* ≤ 0.04) than the C-8h group on d 4 and 5.

Meal size

A source × conditioning × rest (nested in time) effect (*p* < 0.01) was observed for meal size. The C-R-0h group was characterized by a greater meal size (*p* < 0.01) than the N-R-0h group on d 0. The C-A-0h group was characterized by a greater meal size (*p* < 0.01) than the N-A-0h group on d 0. The C-A-8h group was characterized by a greater meal size (*p* < 0.01) than the C-A-0h and N-R-8h group on d 0. The N-A-0h group was characterized by a greater meal size (*p* < 0.01) than the N-A-8h group on d 0. The C-R-8h group was characterized by a greater meal size (*p* < 0.01) than the N-R-8h group on d 0. The C-A-0h group was characterized by a greater meal size (*p* < 0.01) than the N-A-0h and C-A-8h group on d 1. The C-A-8h group was characterized by a greater meal size (*p* < 0.01) than the N-A-8h group on d 1. The N-R-0h and the C-A-0h group were characterized by a greater meal size (*p* ≤ 0.01) than the C-R-0h group on d 3. The C-R-8h group was characterized by a greater meal size (*p* ≤ 0.01) than the C-R-0h group on d 3. The N-R-0h group was characterized by a greater meal size (*p* ≤ 0.01) than the N-A-0h group on d 3. The C-A-8h group was characterized by a greater meal size (*p* < 0.01) than the N-A-8h group on d 3. The C-A-0h group was characterized by a greater meal size (*p* = 0.02) than the N-A-0h group on d 12 and 14. The C-A-8h group was characterized by a greater meal size (*p* = 0.02) than the N-A-8h group on d 18.

Higuchi H., Katoh N., Miyamoto T., Uchida E., Yuasa A. and Takahashi K., 1994. Dexamethasone-induced haptoglobin release by calf liver parenchymal cells. *American Journal of Veterinary Research*, **55**, 1080–1085S1 Fig. Least squares-means of feeding behavior for conditioned (C) or non-conditioned (N), auction market (A) or ranch direct (R) calves rested for 0 (0 h) or 8 (8 h) h.

1. Feeding rate, (B) feeding time, (C) meal frequency, (D) meal size, (E) feeding intake, and (F and G) meal duration.

Higuchi H., Katoh N., Miyamoto T., Uchida E., Yuasa A. and Takahashi K., 1994. Dexamethasone-induced haptoglobin release by calf liver parenchymal cells. *American Journal of Veterinary Research*, **55**, 1080–1085 Higuchi H., Katoh N., Miyamoto T., Uchida E., Yuasa A. and Takahashi K., 1994. Dexamethasone-induced haptoglobin release by calf liver parenchymal cells. *American Journal of Veterinary Research*, **55**, 1080–1085 Higuchi H., Katoh N., Miyamoto T., Uchida E., Yuasa A. and Takahashi K., 1994. Dexamethasone-induced haptoglobin release by calf liver parenchymal cells. *American Journal of Veterinary Research*, **55**, 1080–1085
